# Venous thromboembolism and cancer risk in patients with a history of migraine: a population-based cohort study

**DOI:** 10.1016/j.rpth.2025.103202

**Published:** 2025-09-30

**Authors:** Oscar Rosenkrantz, Dóra K. Farkas, Erzsébet Horváth-Puhó, Søren K. Martiny, Holly Elser, Cecilia H. Fuglsang, Henrik T. Sørensen

**Affiliations:** 1 Department of Clinical Epidemiology and Center for Population Medicine, Department of Clinical Medicine, Aarhus University Hospital and Aarhus University, Aarhus, Denmark; 2 Department of Anesthesiology, Surgery and Trauma Centre, Copenhagen University Hospital–Rigshospitalet, Copenhagen, Denmark; 3 Department of Neurology, Hospital of the University of Pennsylvania, Philadelphia, USA

**Keywords:** Cancer, Denmark, Migraine, Thromboembolism, Cohort Studies

## Abstract

**Background:**

Migraine is associated with elevated risk of venous thromboembolism (VTE). Although unprovoked VTE is recognized as a potential marker of occult cancer, it remains unclear whether VTE in patients with migraine is a marker of cancer.

**Objective:**

To examine cancer risk following VTE in patients with migraine, compared with expected risks in the general population.

**Methods:**

A population-based cohort study using Danish health registry data. Patients with first-time VTE diagnosis and history of migraine (discharge diagnosis or migraine-specific medications) were identified between 1996-2022. Follow-up extended until cancer diagnosis, death, emigration, 15 years, or December 31, 2022. We computed cumulative cancer risk and standardized incidence ratios (SIRs) using national cancer incidence rates during the first year and from 1 to 15 years.

**Results:**

We identified 7131 patients with VTE diagnosis and migraine. Most (71.2%) were identified by prescriptions, and the median follow-up time was 5.4 years. Within the first year, the cumulative cancer risk was 4.4%, SIR 4.28 (95% CI, 3.81-4.78), and lung, pancreatic, and ovarian cancers were observed most frequently when comparing with expected observations from the general population. After first year of follow-up, cumulative risk was 15.6%, and the SIR was 1.15 (95% CI, 1.06-1.25).

**Conclusion:**

Patients with migraine and VTE had elevated short-term and long-term risks of cancer compared with the general population. These findings are in line with other VTE cohorts, suggesting that VTE may be a marker of occult cancer in patients with a history of migraine, particularly within the first year of follow-up.

## Essentials

• Migraine may be associated with an elevated risk of venous blood clots.
• This Danish population-based cohort study examined cancer risk after a venous thrombosis.
• Cancer risk was 4× higher the year after venous thrombosis, despite already elevated venous thrombosis risk with migraine.
• Some cancers, like lung and ovarian, were more common than expected in the first year.

## 1 Introduction

Venous thromboembolism (VTE), encompassing deep venous thrombosis (DVT) and pulmonary embolism (PE), is a well-established complication in patients with cancer [1]. This association is commonly attributed to malignancy-related hypercoagulability, immobility, vascular compression by tumors, and systemic cancer therapies [2,3]. In some cases, VTE may precede the clinical diagnosis of cancer, serving as a potential marker of occult malignancy [[4], [5], [6], [7]]. However, current evidence suggests that the number needed to screen for underlying cancer in the general VTE population is not sufficiently favorable to justify routine, systematic diagnostic work-up [8].

Migraine, a prevalent neurologic disorder affecting more than one billion people globally [9], has been associated with an elevated VTE risk, with adjusted hazard ratios (aHR) ranging from 1.59 (95% CI, 1.45-1.74) in the general migraine population [10] to 3.23 (95% CI, 2.06-7.07) in pregnant patients [11]. Subgroup analyses suggest that this risk is particularly elevated among patients with migraine with aura (aHR, 2.42; 95% CI, 1.40-4.19), while no meaningful association was observed for migraine without aura (aHR, 1.12; 95% CI, 0.92-1.35) [12]. These associations may reflect shared underlying risk factors such as genetic predisposition, hormonal influences, and obesity [13].

Patients with migraine do not appear to have an elevated cancer risk [14] and they are often younger than other first-time VTE populations, which may suggest a lower likelihood of VTE-related cancer. Still, potential mechanisms, including endothelial dysfunction, platelet hyperreactivity, and systemic inflammation, provide a rationale for exploring possible links between migraine pathophysiology and cancer-associated thrombosis. From both clinical and mechanistic perspectives, it is unclear whether patients with migraine who develop VTE have a different cancer risk profile compared with other VTE patients. Moreover, it is unclear whether VTE serves as a marker of occult cancer in this subgroup. To our knowledge, no previous studies have investigated this association.

In this population-based cohort study, we examined the overall and site-specific cancer risks among all patients diagnosed with VTE with a history of migraine compared with expected cancer risks in the Danish general population. Because diagnosis of certain cancers may be delayed, we separately examined cancer risks within the first year and from 1 to 15 years after the initial diagnosis of VTE.

## 2 Methods

### 2.1 Study design and setting

This population-based cohort study was conducted in Denmark from January 1, 1996, to December 31, 2022. The Danish health care system provides universal tax-funded health care to all Danish residents, ensuring universal access to hospitals and private practitioners, and partial reimbursement for prescribed medications [15]. At birth or upon immigration, all Danish residents are assigned a unique 10-digit number by the Danish Civil Registration System. This identifier allows unambiguous individual-level linkage of data among nationwide Danish healthcare registries [16].

The Danish National Patient Registry (DNPR) [17] and the Danish National Prescription Registry [18] were used to create the study cohort. The DNPR contains nationwide data on all nonpsychiatric inpatient admissions, with complete registration beginning in 1978. Outpatient and emergency room visits were recorded beginning in 1995. The registry contains information on the date of contact, the date of discharge, a primary diagnosis, multiple secondary diagnoses (optional), and surgical procedures. Diagnoses were recorded according to the International Classification of Diseases, Eighth Revision (ICD-8) until the end of 1993 and the Tenth Revision (ICD-10) thereafter. The Prescription Registry contains information on all prescription medications dispensed at community pharmacies since 1995 [18]. The Danish Cancer Registry [19] was used to identify incident cancer cases. Established in 1943, this nationwide cancer registry records all new cancer cases, with coding based on the ICD system and the International Classification of Diseases for Oncology.

### 2.2 Study population

Adults aged ≥18 years with a first-time diagnosis of VTE (ie, DVT or PE) were identified from the DNPR using primary and secondary discharge diagnoses assigned during inpatient hospitalizations and outpatient clinic visits. The positive predictive value for VTE coding has been reported to be 93% in the DNPR [20,21]. Diagnoses from emergency department visits were excluded because of their low positive predictive value (31%) [22]. The index date was defined as the date of the first-time diagnosis of VTE.

Among patients with VTE, we identified those with a history of migraine, defined using either a hospital discharge diagnosis of migraine or at least 2 redeemed prescriptions for a migraine-specific medication. Migraine types were categorized based on identification through hospital discharge diagnosis or prescriptions and by aura status (with aura, without aura, other type, no aura status code). Patients fulfilling both criteria, were categorized as identified by diagnosis. Identification through prescription medications was used to capture the majority of migraine patients who were treated solely by general practitioners. At least 2 prescriptions had to be redeemed for any of the following medications: triptans, ergot derivatives, clonidine, or pizotifen, all classified under anatomical therapeutic chemical codes for migraine treatment. Patients with a cancer diagnosis before their index date were excluded. Excepted was nonmelanoma skin cancer due to the high prevalence, low fatality, and inconsistent registration of this cancer type. See Supplementary Table 1 for definitions of diagnosis, anatomical therapeutic chemical codes, and procedural codes used in this study.

Follow-up extended from the index date until a cancer diagnosis, 15 years after the index date, death, emigration, or end of the study period on December 31, 2022, whichever came first.

### 2.3 Cancer outcomes

Information on cancer diagnoses was obtained for cancers registered after the index date in the Danish Cancer Registry. Cancer outcomes included all cancers, except nonmelanoma skin cancer, categorized into cancer groups and site-specific cancer types. Consistent with prior analyses, cancer groups according to likely etiology comprised hormone-related cancers, hematologic cancers, immune-related cancers, gastrointestinal cancers, smoking-related cancers, cancers of neurologic origin, and all other cancers [14]. Information on the cancer stage at the time of diagnosis, categorized as localized, regional spread, metastatic spread, or unknown/missing stage, was also obtained from the cancer registry. For a detailed list of codes used in this study and categorization of cancer types, see Supplementary Table 2.

### 2.4 Covariates

Information on sex (female or male) and age was retrieved from the Danish Civil Registration System. From the DNPR, information was obtained on the calendar year of VTE diagnosis, the presence of at least one classic provoking factor for VTE (ie, major surgery, trauma/fractures, or pregnancy) within 90 days before the index date, and a modified Charlson Comorbidity Index (CCI) score that excluded cancer-specific comorbidities. Based on the CCI score, the comorbidity burden was classified as low (CCI score 0), moderate (CCI score 1-2), or high (CCI score ≥3). Additionally, information on medications prescribed within 1 year before the index date was collected, including nonsteroidal anti-inflammatory drugs (NSAIDs), lipid-lowering drugs, vitamin K antagonists, direct oral anticoagulants, clopidogrel, acetylsalicylic acid, other platelet aggregation inhibitors, combined oral contraceptives, systemic glucocorticoids, and other immunosuppressives. These medications were included as covariates because they may influence the risk of vascular events, inflammation, or hormonal changes potentially relevant to the study outcomes. For details regarding coding and descriptions of covariates and comedications, see Supplementary Tables 2-5.

### 2.5 Statistical analyses

We characterized the cohort according to covariates and migraine type. Categorical variables were presented as counts and percentages, while continuous variables were shown as medians with quartiles (Q1;Q3).

Main analyses. For all patients with VTE and a history of migraine, the Aalen–Johansen estimator was used to compute the cumulative incidence of cancer during the first year and from 1 to 15 years following VTE diagnosis, treating death as a competing risk. Next, to determine the relative cancer risk after VTE, standardized incidence ratios (SIRs) were calculated by dividing observed cancer cases by expected cases. The expected cases were based on national cancer incidence rates stratified by sex, 5-year age categories, and 5-year calendar year intervals of cancer diagnosis. The SIRs were calculated for the first year and from 1 to 15 years after VTE diagnosis. The observed cancer cases were assumed to follow a Poisson distribution when calculating 95% CIs. Exact limits were used when the observed number of cases was fewer than 10; otherwise, Byar’s approximation was used [23].

Subgroup analyses. We computed SIRs and cumulative incidence stratified according to (1) sex (female, male); (2) age at VTE diagnosis (18-39, 40-49, 50-59, 60-69, ≥70 years); (3) calendar year of VTE diagnosis (1996-2000, 2001-2005, 2006-2010, 2011-2016, 2017-2022); (4) VTE type (DVT or PE); (5) presence of provoking factors for VTE; (6) migraine type (migraine identified by prescriptions only, migraine identified by hospital diagnosis); (7) aura status for migraine identified by hospital diagnosis (with aura, without aura, other types, no aura status code [ICD-8]); (8) CCI score; (9) selected comedications, as defined in the covariates section above; (10) cancer stage at diagnosis; and (11) cancer groups and site-specific cancer types as defined under cancer outcomes. Cumulative incidence curves stratified by VTE type and by cancer stage at diagnosis were generated to visualize cancer risk over time in these subgroups.

Secondary Analyses. The number needed to detect one excess cancer was computed as the reciprocal of the excess risk, representing the number of patients with a history of migraine requiring diagnostic evaluation after VTE diagnosis to detect one additional cancer case, under the assumption that cancers diagnosed within 6 months after the index date were present on the date of VTE.

The statistical analyses were performed using the SAS statistical software package, Version 9.4 (SAS Institute Inc).

### 2.6 Ethical standards statement

The study was registered with the Danish Data Protection Agency by Aarhus University (record number 2016-051-000001-0812). According to Danish law, informed consent and approval from ethics committees are not required for registry-based studies.

## 3 Results

A total of 165,895 adults who experienced a first-time VTE between 1996 and 2022 were identified. Of these, 7131 patients (4.3%) with a history of migraine and no previous diagnosis of cancer were included in the study (Supplementary Figure 1). Most (71.2%) were identified by prescriptions for migraine-specific medications, and the rest (28.8%) by hospital discharge diagnosis. The median age at VTE diagnosis was 60.9 years (Q1;Q3, 48.8;72.1), and 77.4% of the patients were females. The median follow-up time was 5.4 years (Q1;Q3, 2.0;10.5). Slightly more than half of identified VTEs were DVT, constituting 56.5% of cases. The comorbidity burden defined by the modified CCI score was low for 53.1% of patients, moderate for 35.8%, and high for 11.1%. Of the patients included, 293 (4.1%) were registered as having migraine with aura (Table 1).

**Table 1:** Baseline characteristics at index date of patients with first-time venous thromboembolism and a history of migraine, Denmark, 1996-2022.

Characteristic	
All, *n* (%)	7131 (100)
Sex, female, *n* (%)	5517 (77.4)
Age, y	
Median (Q1;Q3)	60.9 (48.8; 72.1)
Age group, *n* (%)	
18-39	866 (12.1)
40-49	1072 (15.0)
50-59	1473 (20.7)
60-69	1605 (22.5)
≥70	2115 (29.7)
Year of VTE diagnosis, *n* (%)	
1996-2000	374 (5.2)
2001-2005	766 (10.7)
2006-2010	1213 (17.0)
2011-2016	2112 (29.6)
2017-2022	2666 (37.4)
VTE subtype, *n (%)*	
Deep venous thrombosis	4029 (56.5)
Pulmonary embolism	3102 (43.5)
Follow-up time after VTE diagnosis, y	
Median (Q1;Q3)	5.4 (2.0;10.5)
Provoked VTE, *n* (%)^a^	
Any provoking factor	1618 (22.7)
Major surgery	1194 (16.7)
Major trauma/fracture	668 (9.4)
Pregnancy	97 (1.4)
Migraine, *n* (%)	
Identified by prescriptions only	5080 (71.2)
Identified by hospital diagnosis	2051 (28.8)
Aura status	
Without aura	405 (5.7)
With aura	293 (4.1)
Other types	541 (7.6)
Defined by ICD-8	812 (11.4)
Migraine hospital encounter type, *n (%)*	
Inpatient	1417 (19.9)
Outpatient	634 (8.9)
Charlson Comorbidity Index score, *n (%)*^b^	
Low (0 points)	3787 (53.1)
Moderate (1-2 points)	2550 (35.8)
High (≥3 points)	794 (11.1)

ICD-8, International Classification of Diseases, Eighth Revision; VTE, venous thromboembolism; Q1, first quartile; Q3, third quartile.

a Recorded in the Danish National Patient Registry within 90 days before VTE event (index date).

b Modified Charlson Comorbidity Index, as cancer-specific conditions (ICD-8 codes 140-194, 195-198, 199, 200-203, 204-207, and 275.59 and ICD-10 codes C00-C75, C76-C80, C81-C85, C88, C90, C91-C95, and C96) were excluded from the calculations.

### 3.1 Cancer risk

During the study period, 853 cancer events were observed during the first 15 years following a first-time VTE diagnosis. Of these, 306 occurred within the first year after VTE, yielding an absolute 1-year cumulative cancer risk of 4.4% (Table 2). Compared with the general population, patients with VTE and migraine had a SIR for cancer of 4.28 (95% CI, 3.81-4.78) within the first year of follow-up. From 1 to 15 years of follow-up, the absolute cumulative cancer risk was 15.6%, and the SIR was 1.15 (95% CI, 1.06-1.25).

**Table 2:** Cumulative incidence and standardized incidence ratios for cancer in patients with venous thromboembolism and a history of migraine, Denmark, 1996-2022.

Characteristic	<1-year follow-up			1-15-year follow-up		
	*n*^a^	Risk % (95% CI)	SIR (95% CI)	*n*^a^	Risk % (95% CI)	SIR (95% CI)
All	306	4.4 (3.9-4.8)	4.28 (3.81-4.78)	547	15.6 (14.3-17.0)	1.15 (1.06-1.25)
Sex						
Female	227	4.2 (3.7-4.7)	4.25 (3.71-4.84)	407	14.9 (13.4-16.4)	1.15 (1.04-1.27)
Male	79	5.0 (4.0-6.1)	4.35 (3.45-5.43)	140	18.3 (15.3-21.5)	1.15 (0.97-1.36)
Age group at VTE diagnosis, y						
18-39	5	0.6 (0.2-1.3)	4.40 (1.42-10.25)	12	2.6 (1.3-4.5)	0.69 (0.36-1.20)
40-49	16	1.5 (0.9-2.4)	4.49 (2.57-7.30)	63	11.5 (8.8-14.5)	1.31 (1.00-1.67)
50-59	52	3.6 (2.7-4.6)	5.07 (3.78-6.64)	126	16.6 (13.9-19.6)	1.16 (0.97-1.39)
60-69	102	6.4 (5.3-7.7)	4.99 (4.07-6.06)	174	22.5 (19.2-25.9)	1.11 (0.95-1.29)
≥70	131	6.3 (5.3-7.4)	3.62 (3.03-4.30)	172	21.1 (17.8-24.6)	1.19 (1.02-1.39)
Year of VTE diagnosis						
1996-2000	13	3.5 (1.9-5.7)	4.39 (2.34-7.51)	46	14.0 (10.5-18.0)	1.16 (0.85-1.55)
2001-2005	25	3.3 (2.2-4.7)	4.03 (2.60-5.94)	99	14.4 (11.9-17.1)	1.18 (0.96-1.44)
2006-2010	43	3.5 (2.6-4.7)	3.83 (2.77-5.15)	160	16.2 (13.9-18.7)	1.21 (1.03-1.41)
2011-2016	100	4.7 (3.9-5.7)	4.40 (3.58-5.35)	181	13.1 (10.8-15.5)	1.14 (0.98-1.31)
2017-2022	125	4.9 (4.1-5.7)	4.39 (3.66-5.24)	61	5.1 (3.8-6.6)	1.01 (0.77-1.30)
VTE subtype						
Deep venous thrombosis	111	2.8 (2.3-3.3)	2.84 (2.34-3.42)	342	15.6 (13.9-17.3)	1.15 (1.03-1.28)
Pulmonary embolism	195	6.4 (5.6-7.3)	5.99 (5.18-6.90)	205	15.8 (13.6-18.1)	1.15 (1.00-1.32)
Provoked VTE						
Any provoking factor	63	4.0 (3.1-5.0)	4.02 (3.09-5.14)	125	15.2 (12.6-18.1)	1.19 (0.99-1.42)
No provoking factors	243	4.5 (3.9-5.0)	4.35 (3.82-4.93)	422	15.7 (14.2-17.3)	1.14 (1.03-1.25)
Migraine						
Identified by prescriptions	223	4.5 (3.9-5.0)	4.32 (3.77-4.92)	409	16.6 (15.0-18.3)	1.22 (1.10-1.34)
Identified by hospital diagnosis	83	4.1 (3.3-5.0)	4.17 (3.32-5.16)	138	13.4 (11.2-15.7)	0.99 (0.83-1.17)
Aura status						
Without aura	13	3.3 (1.8-5.4)	4.28 (2.28-7.32)	21	11.8 (6.7-18.5)	1.02 (0.63-1.57)
With aura	14	4.9 (2.8-7.8)	5.95 (3.25-9.99)	18	14.4 (8.0-22.6)	1.49 (0.88-2.36)
Other types	18	3.4 (2.1-5.2)	4.06 (2.40-6.41)	26	9.7 (6.2-14.2)	0.82 (0.54-1.21)
Defined by ICD-8^b^	38	4.7 (3.4-6.3)	3.76 (2.66-5.17)	73	15.1 (12.0-18.6)	0.97 (0.76-1.22)
Charlson Comorbidity Index score						
Low (0)	158	4.2 (3.6-4.9)	4.78 (4.07-5.59)	313	15.7 (14.0-17.5)	1.17 (1.04-1.30)
Moderate (1-2)	115	4.6 (3.8-5.5)	4.00 (3.30-4.80)	185	16.6 (14.1-19.2)	1.12 (0.96-1.29)
High (≥3)	33	4.2 (3.0-5.8)	3.38 (2.32-4.74)	49	12.6 (9.2-16.5)	1.19 (0.88-1.57)

ICD-8, International Classification of Diseases, Eighth Revision; SIR, standardized incidence ratio; VTE, venous thromboembolism.

a Observed cases.

b No aura status coding in the ICD-8.

### 3.2 Subgroup analysis

Stratification by age showed absolute risks elevated with age, but SIRs in the first year of follow-up were similar to the overall estimate across all age groups. From 1 to 15 years of follow-up, SIRs across age groups varied slightly. However, the confidence intervals were wide, and the results did not allow for firm conclusions regarding specific age groups. Relative risks were broadly similar between males and females (Table 2).

The absolute 1-year cumulative cancer risk was substantially higher among patients with PE (6.4%; 95% CI, 5.6%-7.3%) compared with patients with DVT (2.8%; 95% CI, 2.3%-3.3%). This difference in cumulative cancer risk between patients with PE and DVT generally seemed to reflect differences within the first months after VTE diagnosis, indicated by the early separation of the cumulative incidence curves with subsequent parallel and steady increases (Figure A). SIRs were elevated in the first year for both patients with PE (5.99; 95% CI, 5.18-6.90) and those with DVT (2.84; 95% CI, 2.34-3.42). The SIRs were attenuated but remained elevated in both subgroups when follow-up extended beyond the first year (PE, 1.15; 95% CI, 1.00-1.32; DVT, 1.15; 95% CI, 1.03-1.28) (Table 2).

**Figure:**
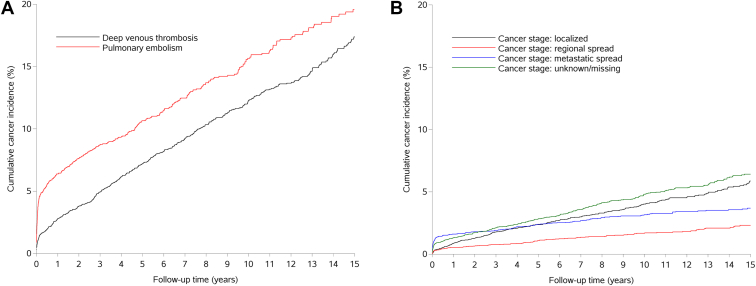
**Cumulative incidence of any cancer after venous thromboembolism in patients with a history of migraine, Denmark, 1996-2022.** (A) Stratified by venous thromboembolism type (deep venous thrombosis or pulmonary embolism). (B) Stratified by cancer stage at diagnosis (localized, regional spread, metastatic spread, or unknown/missing stage)

During the first year of follow-up, SIRs were elevated across migraine identification types, ie, prescriptions (4.32; 95% CI, 3.77-4.92) and hospital diagnoses (4.17; 95% CI, 3.32-5.16), and aura status subgroups, with values ranging from 3.76 (95% CI, 2.66-5.17) for cases defined by ICD-8 to 5.95 (95% CI, 3.25-9.99) for patients with migraine with aura (Table 2). However, confidence intervals again were wide, and no consistent differences between migraine types were observed. Beyond the first year of follow-up (1 to 15 years), SIRs declined in all groups, with estimates close to one and overlapping confidence intervals across subgroups.

In analyses stratified by comedication, most groups had one-year cumulative cancer risks and SIRs similar to those for the overall cohort. For patients using combined oral contraceptives, the cumulative cancer risk was 4.3% (95% CI, 2.4%-7.0%) during the follow-up period beyond the first year (1 to 15 years), and the SIR was 0.65 (95% CI, 0.35-1.09) for the same period. On the other end of the spectrum, patients taking immunosuppressive medication within 1 year before the index date had a cumulative cancer risk of 33.6% (95% CI, 21.4%-46.3%) during the follow-up period beyond the first year (1 to 15 years), and the SIR was 2.43 (95% CI, 1.59-3.57) (Supplementary Table 6).

Analyses stratified by calendar year of VTE diagnosis, provoking factors, and comorbidity burden (CCI) showed no substantial deviations from the main analysis. Detailed results are available in Table 2.

The SIRs within the first year of follow-up for cancer stage at diagnosis increased with advancing cancer: 2.40 (95% CI, 1.84-3.09) for localized cancer, 3.18 (95% CI, 2.25-4.36) for regional spread, and 9.17 (95% CI, 7.56-11.01) for metastatic cancer (Table 3). The cumulative incidence plot (Figure B) shows that metastatic cancers had the highest cumulative incidence during the first year, followed by cancers with unknown stage, while localized and regional spread cancers had the lowest. However, after the first year, cancers with unknown stage and localized cancers had the highest cumulative incidence over time, followed by metastatic cancers. Cancers with regional spread had the lowest cumulative incidence throughout the 15-year follow-up period.

**Table 3:** Standardized incidence ratios for cancer by cancer group and site-specific cancer type after a first-time venous thromboembolism diagnosis in patients with a history of migraine, Denmark, 1996-2022.

Characteristic	<1-year follow-up			1-15-year follow-up		
	*n*^a^	Risk % (95% CI)	SIR (95% CI)	*n*^a^	Risk % (95% CI)	SIR (95% CI)
All cancers	306	4.4 (3.9-4.8)	4.28 (3.81-4.78)	547	15.6 (14.3-17.0)	1.15 (1.06-1.25)
Hormone-related cancers	63	0.9 (0.7-1.1)	2.69 (2.06-3.44)	173	4.7 (4.0-5.5)	1.11 (0.95-1.29)
Breast	22	0.3 (0.2-0.5)	1.52 (0.95-2.30)	112	3.2 (2.6-3.9)	1.16 (0.96-1.40)
Uterus	—	0.1 (0.1-0.2)	3.52 (1.61-6.68)	14	0.4 (0.2-0.7)	0.84 (0.46-1.40)
Ovary	17	0.2 (0.1-0.4)	9.87 (5.75-15.80)	14	0.4 (0.2-0.7)	1.28 (0.70-2.14)
Prostate	14	0.2 (0.1-0.3)	3.05 (1.67-5.11)	33	0.8 (0.5-1.1)	1.05 (0.72-1.48)
Testicle	<5	—	—	0	—	—
Gastrointestinal cancers	38	0.5 (0.4-0.7)	3.03 (2.14-4.16)	83	2.5 (1.9-3.1)	1.00 (0.80-1.24)
Esophagus	<5	—	—	<5	—	—
Stomach	<5	—	—	5	0.2 (0.1-0.4)	0.72 (0.23-1.67)
Small intestine	<5	—	—	<5	—	—
Colorectal	25	0.4 (0.2-0.5)	2.72 (1.76-4.01)	56	1.6 (1.2-2.1)	0.93 (0.70-1.20)
Liver and intrahepatic bile duct	5	0.1 (0.0-0.2)	6.87 (2.23-16.02)	10	0.3 (0.1-0.5)	2.02 (0.97-3.71)
Gallbladder and biliary tree	<5	—	—	7	0.2 (0.1-0.4)	2.15 (0.86-4.44)
Smoking-related cancers	131	1.9 (1.6-2.2)	7.04 (5.88-8.35)	145	4.1 (3.4-4.8)	1.18 (0.99-1.39)
Tongue	0	—	—	<5	—	—
Oral cavity	0	—	—	<5	—	—
Tonsil and oropharynx	0	—	—	<5	—	—
Pancreas	23	0.3 (0.2-0.5)	10.94 (6.93-16.41)	22	0.6 (0.4-0.9)	1.59 (0.99-2.40)
Lung, bronchi, and trachea	91	1.3 (1.0-1.6)	9.54 (7.68-11.71)	78	2.2 (1.7-2.8)	1.24 (0.98-1.54)
Kidney	9	0.1 (0.1-0.2)	6.54 (2.99-12.42)	14	0.4 (0.2-0.6)	1.52 (0.83-2.56)
Bladder	<5	—	—	16	0.5 (0.3-0.9)	0.78 (0.45-1.27)
Thyroid	<5	—	—	5	0.1 (0.1-0.4)	1.23 (0.40-2.86)
Cancer of neurologic origin	10	0.1 (0.1-0.3)	3.19 (1.53-5.87)	29	0.9 (0.6-1.2)	1.37 (0.92-1.97)
Brain	5	0.1 (0.0-0.2)	3.55 (1.15-8.26)	—	0.3 (0.1-0.6)	0.97 (0.45-1.85)
Membrane of the brain and spinal meninges	<5	—	—	16	0.4 (0.3-0.7)	1.82 (1.04-2.95)
Other (central/peripheral nervous system)	<5	—	—	<5	—	—
Hematologic cancers	40	0.6 (0.4-0.8)	6.22 (4.44-8.47)	50	1.4 (1.0-1.9)	1.18 (0.88-1.56)
Metastases, nonspecified in lymph nodes	11	0.2 (0.1-0.3)	9.11 (4.54-16.30)	10	0.3 (0.1-0.5)	1.37 (0.65-2.51)
Hodgkin lymphoma	<5	—	—	<5	—	—
Non–Hodgkin lymphoma	18	0.3 (0.2-0.4)	7.39 (4.38-11.68)	20	0.6 (0.3-0.9)	1.22 (0.75-1.89)
Multiple myeloma	<5	—	—	8	0.2 (0.1-0.5)	1.25 (0.54-2.46)
Lymphoid leukemia	<5	—	—	7	0.2 (0.1-0.4)	1.09 (0.44-2.25)
Myeloid leukemia	5	0.1 (0.0-0.2)	9.14 (2.96-21.29)	<5	—	—
Monocytic leukemia	<5	—	—	0	—	—
Immune-related cancers	14	0.2 (0.1-0.3)	2.49 (1.36-4.18)	46	1.5 (1.1-2.0)	1.21 (0.89-1.61)
Anal canal	<5	—	—	<5	—	—
Malignant melanoma	8	0.1 (0.1-0.2)	1.98 (0.86-3.91)	36	1.1 (0.8-1.6)	1.30 (0.91-1.79)
External female genitalia	<5	—	—	<5	—	—
Cervix	<5	—	—	6	0.2 (0.1-0.5)	1.18 (0.43-2.57)
All other cancers	10	0.1 (0.1-0.3)	5.65 (2.71-10.40)	21	0.6 (0.4-0.9)	1.84 (1.14-2.81)
Cancer stage						
Localized stage	61	0.9 (0.7-1.1)	2.40 (1.84-3.09)	192	5.6 (4.8-6.5)	1.15 (0.99-1.32)
Regional spread stage	38	0.5 (0.4-0.7)	3.18 (2.25-4.36)	68	2.0 (1.5-2.6)	0.91 (0.70-1.15)
Metastatic spread stage	114	1.6 (1.3-1.9)	9.17 (7.56-11.01)	87	2.3 (1.8-2.9)	1.08 (0.86-1.33)
Unknown or missing stage	93	1.3 (1.1-1.6)	4.27 (3.44-5.23)	200	5.7 (4.9-6.6)	1.31 (1.14-1.51)

Cells with <5 outcomes and those allowing back-calculation of these values have been removed for anonymization. “—” denotes that SIR and CI could not be calculated due to too few events.

SIR, standardized incidence ratio; VTE, venous thromboembolism.

a Observed cases.

During the first year of follow-up, cancer risk was substantially elevated compared with the general population. The most frequently observed cancer groups were smoking-related (*n* = 131; SIR, 7.04; 95% CI, 5.88-8.35), hematologic (*n* = 40; SIR, 6.22; 95% CI, 4.44-8.47), gastrointestinal (*n* = 38; SIR, 3.03; 95% CI, 2.14-4.16), and hormone-related cancers (*n* = 63; SIR, 2.69; 95% CI, 2.06-3.44). At the site-specific level, lung (*n* = 91; SIR, 9.54; 95% CI, 7.68-11.71), pancreatic (*n* = 23; SIR, 10.94; 95% CI, 6.93-16.41), and colorectal cancers (*n* = 25; SIR, 2.72; 95% CI, 1.76-4.01) were most frequent. Beyond the first year (1 to 15 years), the elevated risk persisted across most cancer groups, although it was attenuated. For gastrointestinal cancers, no excess risk was observed during long-term follow-up (SIR, 1.00; 95% CI, 0.80-1.24). Similarly, cancers of neurologic origin did not clearly show persistent elevated relative risk (SIR, 1.37; 95% CI, 0.92-1.97). Generally, estimates beyond the first year were accompanied by wide confidence intervals. See Table 3 for full results.

### 3.3 Secondary analyses

Even with the high SIR observed during the first year of follow-up, approximately 34 (95% CI, 31-38) patients with migraine and VTE would need to have diagnostic work-up done to detect one excess cancer within the first 6 months after VTE, based on the 248 cancers observed and 37 expected within 6 months of follow-up.

## 4 Discussion

In this population-based cohort study including only patients with a first-time VTE and a history of migraine, we observed an elevated risk of first-time cancer diagnoses compared with the general population. Within the first year of follow-up, patients with migraine had a 4.4% absolute cancer risk after a VTE diagnosis, and the corresponding relative risk was 4 times higher compared with the general population. The risk was particularly high for cancers diagnosed at an advanced stage. From 1 to 15 years of follow-up, the relative cancer risk declined but remained elevated at a point estimate of excess risk at 15%.

Similar trends showing elevated cancer risk in patients with VTE, particularly in the first year of follow-up, have been reported in studies focusing on other patient groups with specific co-existing conditions, such as diabetes [24], diverticular disease [25], and dementia [26]. One-year absolute risks ranged from 3.1% in patients with inflammatory bowel disease [27] (SIR, 3.2; median follow-up 5.9 years) to 6.7% in low-dose aspirin users [28] (SIR, 2.8-3.3; median follow-up 2.2 years), with median follow-up times generally spanning 1 to 4 years [[24], [25], [26], [27], [28], [29], [30], [31], [32]]. We have not identified previous studies examining the cancer risk after VTE in patients with a history of migraine.

The cancer risk following VTE was broadly consistent across most subgroups of patients with a history of migraine. SIRs in the first year were similar to the overall cohort across sex and age groups, although absolute risks increased with advancing age, in alignment with previous studies [24,31].

The elevated cancer risk associated with PE compared with DVT, particularly during the first year after VTE, may reflect the finding that PE is more commonly linked to malignancies and thus might act as a stronger marker of occult cancer. Although earlier studies support the stronger association of PE with occult cancer, definitive evidence remains limited [6,33]. Heightened diagnostic work-up also may influence the association of PE with cancer, as more extensive diagnostic investigation in these patients may lead to more frequent detection of underlying cancer compared with patients with deep vein thrombosis. However, the long-term parallel incidence curves suggest that this difference in early detection does not reflect a compensatory diagnostic deficit in DVT patients, as there is no subsequent catch-up in cancer diagnoses over time.

Across migraine types, including those with aura, SIRs in the first year of follow-up were elevated and aligned with the main analysis. There was no clear difference between types, suggesting that the underlying migraine phenotype may not substantially influence the observed cancer risk, consistent with prior evidence indicating uniform comorbidity risks across migraine subtypes [10]. However, small numbers limited interpretation. Similarly, stratification by comorbidity burden, provoking factors, method of migraine identification, and calendar year of VTE diagnosis showed no substantial deviation from the main analysis, indicating consistency of the association across these groups, as observed in previous VTE cohort studies [30].

When we stratified the analysis by comedications, the group of patients receiving contraceptives had a lower absolute cancer risk following VTE and a low SIR from 1 to 15 years of follow-up, compared with previous findings [34]. This may be attributable to selection bias or a healthier baseline profile in contraceptive users, as this group generally comprises younger, healthier patients. Patients using immunosuppressive therapy had a substantially higher cancer risk, suggesting that immunosuppression may modify the association between VTE and cancer.

The strong association between VTE and metastatic cancer suggests that occult cancer, particularly advanced-stage cancer, is a major contributor to VTE in these patients. Advanced cancers are often associated with a hypercoagulable state and vascular compression by the tumor, which increases the likelihood of thrombotic events [35,36].

Smoking-related cancers, particularly lung cancer and hematologic cancers, showed notably elevated SIRs in the first year after VTE, consistent with other studies highlighting the role of thrombotic events as early markers of occult cancer [6,37,38]. The high risk of smoking-related cancers may reflect shared risk factors, such as smoking [39], which predispose patients with a history of migraine to both cancer and VTE. However, the effect of shared behavioral risk factors in patients with migraine, or broader systemic processes linking migraine, VTE, and cancer, remains uncertain.

In a Danish cohort study on migraine and cancer risk [14], showed a slightly elevated risk of neurologic cancers and a decreased risk of gastrointestinal cancers. In the current study, during the follow-up period beyond the first year (1 to 15 years), gastrointestinal cancers had the lowest risk compared with the general population among all cancer groups, though confidence intervals were wide. This lower risk may be due to the protective effects of NSAIDs and aspirin, which are frequently used in migraine management and have been associated with reduced risk of gastrointestinal cancers [40,41]. However, the risk of neurologic cancers was not clearly persistently elevated over the long term, despite previous descriptions of headaches serving as an early symptom of undiagnosed tumors in the brain and surrounding membranes, as suggested elsewhere [42].

Previous studies have not shown extensive screening following VTE to be beneficial [[43], [44], [45], [46], [47]]. The elevated cancer risk seen shortly after VTE diagnosis appears to be driven primarily by occult cancers diagnosed within the first months after VTE. Our findings suggest that this also applies to the subgroup of patients with both migraine and VTE. Despite a high SIR during the first year of follow-up, the absolute excess risk remains modest. Based on 248 cancers observed versus 37 expected within the first 6 months, approximately 34 (95% CI, 31-38) patients with migraine and VTE would need to undergo diagnostic work-up to detect one excess cancer. Therefore, based on current evidence, limited screening based on patient history and physical examination remains the recommended strategy [[47], [48], [49]]. This also applies to this subgroup of patients with migraine, among whom the absolute excess risk, despite a high SIR, remained modest.

### 4.1 Study strengths and limitations

This was a nationwide population-based study with complete follow-up, reducing the risk of referral and selection bias. Further, the positive predictive values of diagnosis coding in the DNPR for the comorbidities [50] and VTE [20], and for cancer in the Danish Cancer Registry [19] have been assessed as overall high for most of the study period. Several limitations should be considered when interpreting our findings. One concern was detection bias, ie, the possibility of increased diagnostic effort, which should be considered when looking at the association between VTE and subsequent cancer during short-term follow-up. However, no compensatory deficit was consistently observed, and the persisting elevated relative risk beyond the first year (1-15 years) after VTE reduces this concern. Another issue was the limited categorization of migraine type, as most patients with migraine were identified by prescriptions only. We were not able to directly compare cancer risk between patients with and without migraine and therefore cannot determine whether migraine per se modifies the association between VTE and cancer. Despite this limitation, prior studies have shown that VTE is associated with an elevated cancer risk in both unprovoked and provoked cases, while migraine is linked to elevated VTE risk but not to elevated cancer risk. Our study, therefore, focused on a previously unexamined subgroup, patients with both migraine and VTE, and our findings suggest that migraine does not modify the established VTE-cancer association, as the observed cancer risk was consistent with that reported in other VTE cohorts. Further, less severe cases of migraine may not be captured, and excluding such cases might have introduced selection bias. However, the direction in which this would influence the results is unclear. The increased number of VTE patients in the most recent study periods can most likely be attributed to an increased use of diagnostic procedures, such as ultrasound and CT scans, and the lack of prescription data prior to 1995. Most patients with migraine were identified through prescription data available from 1995 onward. This may have led to an underestimation of migraine prevalence in the early study period due to underregistration of migraine. Some patients with migraine have used NSAIDs or low-dose aspirin regularly, which has been associated with a reduced cancer risk. This could potentially confound the association between migraine and cancer. Also, some site-specific results for cancer should be interpreted with caution, as some cancer sites had few observations and, thus, reduced the robustness of the results. Stratified analyses by provoking factors, migraine diagnosis method, comorbidity burden, and calendar year of VTE diagnosis did not reveal substantial variation from the main findings, reducing concern about residual confounding in these subgroups.

## 5 Conclusion

Patients with a history of migraine and VTE had elevated short-term and long-term risks of cancer compared with the general population. These findings are in line with observations in other VTE cohorts, suggesting that VTE may be a marker of occult cancer in patients with a history of migraine, particularly within the first year of follow-up.
