# Supplementary material for: Venous thromboembolism and cancer risk in patients with a history of migraine: a population-based cohort study
Source: Res Pract Thromb Haemost. 2025 Sep 30;9(7):103202. doi: 10.1016/j.rpth.2025.103202 (PMC12594897; doi:10.1016/j.rpth.2025.103202)
Supplement: Supplementary Material [file mmc1.docx]

**Online supplemental material**

**Title:**

Venous thromboembolism and cancer risk in patients with a history of migraine: a population-based cohort study

**Journal:**

Research and Practice in Thrombosis and Haemostasis

**Correspondence:**

Oscar Rosenkrantz

Department of Clinical Epidemiology, Aarhus University Hospital and Aarhus University

Olof Palmes Allé 43, 45, DK, Aarhus N, 8200, Denmark

Tel +45 87 16 72 12

Email [oscar.oelrich.rosenkrantz@clin.au.dk](mailto:oscar.oelrich.rosenkrantz@clin.au.dk)

Contents

[**TABLE S1.** Definition of exposures 2](#_Toc207165046)

[**TABLE S2.** Definition of cancer outcomes 3](#_Toc207165047)

[**TABLE S3.** Definition of comedications 4](#_Toc207165048)

[**TABLE S4.** Definition of venous thromboembolic provoking factors 5](#_Toc207165049)

[**TABLE S5.** Definition of Charlson Comorbidity Index diagnoses 6](#_Toc207165050)

[**TABLE S6.** The cumulative incidence and standardized incidence ratios of cancer in patients with venous thromboembolism and a history of migraine, stratified on comedications, Denmark, 1996–2022 7](#_Toc207165051)

[**FIGURE S1.** Flowchart of included study participants 8](#_Toc207165052)

### **TABLE S1.** Definition of exposures

| **Exposures** | **ICD-8 codes** | **ICD-10 codes** | **Type of contact and diagnosis** | **ATC codes** |
| --- | --- | --- | --- | --- |
| **Migraine diagnosis** |  |  |  |  |
| Migraine total | 346.00  346.08  346.09 | G43.0–43.3 (excluding G43.3A: migraine with cerebral infarction),  G43.8  G43.9 | Inpatient and outpatient, primary and secondary diagnoses | — |
| Migraine with aura | — | G43.1 | Inpatient and outpatient, primary and secondary diagnoses | — |
| Migraine without aura | — | G43.0 | Inpatient and outpatient, primary and secondary diagnoses | — |
| **Migraine prescription** |  |  |  |  |
| Migraine identified by prescriptions | — | — | At least two redeemed prescriptions. | N02CC, N02CA01–02,  N02CA04, N02CA52  N02CX01–02 |
| **VTE** | 451.00  450.99 | I80.1–3  I26 | Inpatient and outpatient, primary and secondary diagnoses. Index date was date of first VTE diagnosis. | — |
| Deep vein thrombosis | 451.00 | I80.1–3 | Inpatient and outpatient, primary and secondary diagnoses | — |
| Pulmonary embolism | 450.99 | I26 | Inpatient and outpatient, primary and secondary diagnoses | — |
| **For exclusion from cohort** | | | | |
| **Cancer history** | — | — | Any cancer^a^ diagnosis, defined as a diagnosis in the Danish Cancer Registry prior to index date | — |

Abbreviations: ATC, Anatomical Therapeutic Chemical classification system; ICD, International Classification of Diseases, Eight or Tenth Revision; VTE, venous thromboembolism

^a^ Except non-melanoma skin cancer

### **TABLE S2.** Definition of cancer outcomes

| **Outcomes** | **ICD-10 codes** | **Type of contact and diagnosis** |
| --- | --- | --- |
| All cancers | C00–C96 (excluding C44), D09.0, D09.5, D30.3, D32, D33.0–D33.2, D33.3–D33.9, D35.2–D35.4, D41.4, D42, D43.0–D43.2, D43.3–D43.9, D44.3–D44.5  These “D” codes from the list above are restricted to morphology codes 812 and 813: D09.0, D.09.5, D30.3, D41.4^a^ | Diagnosis (any) after the index |
| **Hormone-related cancers** |  |  |
| Breast | C50 | Diagnosis (any) after the index |
| Uterus | C54–55 | Diagnosis (any) after the index |
| Ovary | C56, C57.0–4 | Diagnosis (any) after the index |
| Prostate | C61 | Diagnosis (any) after the index |
| Testicle | C62 | Diagnosis (any) after the index |
| **Gastrointestinal cancers** |  |  |
| Esophagus | C15 | Diagnosis (any) after the index |
| Stomach | C16 | Diagnosis (any) after the index |
| Small intestine | C17 | Diagnosis (any) after the index |
| Colorectal | C18–20 | Diagnosis (any) after the index |
| Liver and intrahepatic bile duct | C22 | Diagnosis (any) after the index |
| Gallbladder and biliary tree | C23–24 | Diagnosis (any) after the index |
| **Smoking-related cancers** |  |  |
| Tongue | C01–02 | Diagnosis (any) after the index |
| Oral cavity | C03–06 | Diagnosis (any) after the index |
| Tonsil and oropharynx | C09–10 | Diagnosis (any) after the index |
| Pancreas | C25 | Diagnosis (any) after the index |
| Lung, bronchi, and trachea | C33–34 | Diagnosis (any) after the index |
| Kidney | C64 | Diagnosis (any) after the index |
| Bladder | C67, D09.0, D09.5, D30.3, and D41.4 (with morphology codes 812 and 813) | Diagnosis (any) after the index |
| Thyroid | C73 | Diagnosis (any) after the index |
| **Cancer of neurological origin** |  |  |
| Brain | C71, C75.1–C75.3, D33.0–D33.2, D35.2–D35.4, D43.0–D43.2, D44.3–D44.5 | Diagnosis (any) after the index |
| Membrane of the brain and spinal meninges | C70, D32, D42 | Diagnosis (any) after the index |
| Other (central and peripheral nervous system) | C47, C72, D33.3–D33.9, D43.3–D43.9 | Diagnosis (any) after the index |
| **Hematologic cancers** |  |  |
| Metastases, non-specified in lymph nodes | C77–79 | Diagnosis (any) after the index |
| Hodgkin lymphoma | C81 | Diagnosis (any) after the index |
| Non-Hodgkin lymphoma | C82–86 | Diagnosis (any) after the index |
| Multiple myeloma | C90 | Diagnosis (any) after the index |
| Lymphoid leukemia | C91 | Diagnosis (any) after the index |
| Myeloid leukemia | C92 | Diagnosis (any) after the index |
| Monocytic leukemia | C93 | Diagnosis (any) after the index |
| **Immune-related cancers** |  |  |
| Anal canal | C21 | Diagnosis (any) after the index |
| Malignant melanoma | C43 | Diagnosis (any) after the index |
| External female genitalia | C51 | Diagnosis (any) after the index |
| Cervix | C53 | Diagnosis (any) after the index |

Abbreviations: ICD, International Classification of Diseases, Tenth Revision

^a^ Thus excluding carcinoma in situ of the breast, dysplasia and carcinoma in situ of cervix uteri, mola and neoplasma placenta, polycythemia vera, myelodysplastic syndromes, and other not-otherwise-specified neoplasms of lymphoid and haematopoetic tissue.

### **TABLE S3.** Definition of comedications

| **Prescriptions** | **ATC codes** |
| --- | --- |
| NSAIDs | M01A |
| Vitamin K antagonists | B01AA |
| Clopidogrel | B01AC04 |
| Acetylsalicylic acid | B01AC06, N02BA01 |
| Other platelet aggregation inhibitors | B01AC01–03, B01AC05, B01AC07–56 |
| DOACs | B01AE, B01AF |
| Lipid-lowering drugs | C10 |
| Combined oral contraceptives | G03AA, G03AB |
| Systemic glucocorticoids | H02AB |
| Immunosuppressive agents | L04 |

Abbreviations: ATC, Anatomical Therapeutic Chemical classification system; DOAC, Direct oral anticoagulant; NSAID, Non-steroidal anti-inflammatory drug

### **TABLE S4.** Definition of venous thromboembolic provoking factors

|  | **ICD-10 codes** | **Notes** |
| --- | --- | --- |
| Pregnancy | O00–O99 | <90 days before index |
| Fractures/trauma | S00–T14 | <90 days before index |
| Surgery | Danish Classification of Surgical Procedures: all codes (00000–99960)  NOMESCO Classification of surgical Procedures: KA–KQ, KX, KY | <90 days before index |

Abbreviations: ICD, International Classification of Diseases, Tenth Revision; NOMESCO, Nordic Medico-Statistical Committee

### **TABLE S5.** Definition of Charlson Comorbidity Index diagnoses

|  | **ICD-8 codes** | **ICD-10 codes** | **Score in Index** |
| --- | --- | --- | --- |
| Myocardial infarction | 410 | I21, I22, I23 | 1 |
| Heart failure | 427.09, 427.10, 427.11, 427.19, 428.99, 782.49 | I50, I11.0, I13.0, I13.2 | 1 |
| Congestive heart failure | 427.09, 427.10, 427.11, 427.19, 428.99, 782.49 | I50, I11.0, I13.0, I13.2 | 1 |
| Peripheral vascular disease | 440, 441, 442, 443, 444, 445 | I70, I71, I72, I73, I74, I77 | 1 |
| Cerebrovascular disease | 430, 431, 433–434 | I60–I69, G45, G46 | 1 |
| Dementia | 290.09–290.19, 293.09 | F00–F03, F05.1, G30 | 1 |
| Chronic pulmonary disease | 490–493, 515–518 | J40–J47, J60–J67, J68.4, J70.1,  J70.3, J84.1, J92.0, J96.1, J98.2, J98.3 | 1 |
| Connective tissue disease | 712, 716, 734, 446, 135.99 | M05, M06, M08, M09, M30, M31,  M32, M33, M34, M35, M36, D86 | 1 |
| Ulcer disease | 530.91, 530.98, 531–534 | K22.1, K25–K28 | 1 |
| Mild liver disease | 571, 573.01, 573.04 | B18, K70.0–K70.3, K70.9, K71, K73, K74, K76.0 | 1 |
| Diabetes | 249.00, 249.06, 249.07, 249.09, 250.00, 250.06, 250.07, 250.09 | E10.0, E10.1, E10.9, E11.0, E11.1, E11.9 | 1 |
| Hemiplegia | 344 | G81, G82 | 2 |
| Moderate to severe renal disease | 403, 404, 580–583, 584, 590.09, 593.19, 753.10–753.19, 792 | I12, I13, N00–N05, N07, N11, N14, N17–N19, Q61 | 2 |
| Diabetes with end-organ damage | 249.01–249.05, 249.08, 250.01–250.05, 250.08 | E10.2–E10.8, E11.2–E11.8 | 2 |
| Any tumor | 140–194 | C00–C75 | 2 |
| Leukemia | 204–207 | C91–C95 | 2 |
| Lymphoma | 200–203, 275.59 | C81–C85, C88, C90, C96 | 2 |
| Moderate to severe liver disease | 070.00, 070.02, 070.04, 070.06, 070.08, 573.00, 456.00–456.09 | B15.0, B16.0, B16.2, B19.0, K70.4, K72, K76.6, I85 | 3 |
| Metastatic solid tumor ^a^ | 195–198, 199 | C76–C80 | 6 |
| AIDS | 079.83 | B21–B24 | 6 |

Abbreviations: AIDS, Acquired Immunodeficiency Syndrome; ICD, International Classification of Diseases, Eighth or Tenth Revision

^a^ Cancer-specific conditions (ICD-8 codes 140–194, 195–198, 199, 200–203, 204–207, 275.59 and ICD-10 codes C00–C75, C76–C80, C81–C85, C88, C90, C91–C95, C96) were not included in the modified Charlson Comorbidity Index calculations.

### **TABLE S6.** The cumulative incidence and standardized incidence ratios of cancer in patients with venous thromboembolism and a history of migraine, stratified on comedications, Denmark, 1996–2022

|  | | **0–1-year follow-up** | | | | **>1–15-year follow-up** | |
| --- | --- | --- | --- | --- | --- | --- | --- |
|  |  | **n^a^** | **Risk %**  **(95% CI)** | **SIR (95% CI)** | **n^a^** | **Risk %**  **(95% CI)** | **SIR (95% CI)** |
| **All** | | 306 | 4.4 (3.9–4.8) | 4.28 (3.81–4.78) | 547 | 15.6 (14.3–17.0) | 1.15 (1.06–1.25) |
| **Comedication** | |  |  |  |  |  |  |
| NSAIDs: no | | 192 | 4.6 (4.0–5.3) | 4.38 (3.78–5.04) | 290 | 15.1 (13.3–16.9) | 1.11 (0.99–1.25) |
| NSAIDs: yes | | 114 | 3.9 (3.3–4.7) | 4.11 (3.39–4.94) | 257 | 16.2 (14.3–18.2) | 1.20 (1.06–1.35) |
| Lipid-lowering drugs: no | | 229 | 4.1 (3.6–4.7) | 4.45 (3.89–5.06) | 427 | 14.8 (13.4–16.2) | 1.15 (1.04–1.26) |
| Lipid-lowering drugs: yes | | 77 | 5.2 (4.2–6.4) | 3.83 (3.02–4.79) | 120 | 21.1 (16.8–25.7) | 1.16 (0.96–1.39) |
| Vitamin K antagonists: no | | 294 | 4.4 (3.9–4.9) | 4.28 (3.80–4.79) | 524 | 15.7 (14.3–17.1) | 1.16 (1.06–1.26) |
| Vitamin K antagonists: yes | | 12 | 4.3 (2.3–7.1) | 4.24 (2.19–7.41) | 23 | 14.4 (8.9–21.2) | 1.08 (0.68–1.61) |
| DOAC: no | | 287 | 4.3 (3.8–4.8) | 4.27 (3.79–4.80) | 534 | 15.6 (14.3–17.0) | 1.15 (1.06–1.26) |
| DOAC: yes | | 19 | 5.3 (3.3–8.0) | 4.33 (2.61–6.77) | 13 | 20.0 (5.3–41.5) | 1.11 (0.59–1.90) |
| Clopidogrel: no | | 290 | 4.4 (3.9–4.9) | 4.40 (3.91–4.94) | 521 | 15.6 (14.2–17.0) | 1.15 (1.06–1.26) |
| Clopidogrel: yes | | 16 | 4.1 (2.5–6.5) | 2.80 (1.60–4.54) | 26 | 15.4 (9.7–22.4) | 1.13 (0.74–1.65) |
| Acetylsalicylic acid: no | | 244 | 4.2 (3.7–4.7) | 4.44 (3.90–5.03) | 429 | 15.2 (13.8–16.7) | 1.14 (1.03–1.25) |
| Acetylsalicylic acid: yes | | 62 | 5.1 (4.0–6.5) | 3.74 (2.87–4.79) | 118 | 17.4 (14.4–20.6) | 1.20 (1.00–1.44) |
| Other platelet aggregation inhibitors: no | | 297 | 4.3 (3.9–4.8) | 4.27 (3.80–4.79) | 534 | 15.6 (14.3–17.0) | 1.15 (1.06–1.25) |
| Other platelet aggregation inhibitors: yes | | 9 | 6.0 (3.0–10.7) | 4.36 (2.00–8.28) | 13 | 14.8 (7.4–24.6) | 1.12 (0.60–1.91) |
| Combined oral contraceptives: no | | 300 | 4.6 (4.1–5.1) | 4.27 (3.80–4.79) | 532 | 17.1 (15.6–18.6) | 1.18 (1.08–1.28) |
| Combined oral contraceptives: yes | | 6 | 1.1 (0.5–2.3) | 4.38 (1.61–9.54) | 14 | 4.3 (2.4–7.0) | 0.65 (0.35–1.09) |
| Systemic glucocorticoids: no | | 254 | 4.2 (3.8–4.8) | 4.27 (3.76–4.83) | 451 | 15.2 (13.8–16.7) | 1.11 (1.01–1.22) |
| Systemic glucocorticoids: yes | | 52 | 5.0 (3.8–6.4) | 4.28 (3.20–5.61) | 96 | 18.0 (14.6–21.7) | 1.41 (1.14–1.72) |
| Other immunosuppresive: no | | 297 | 4.3 (3.9–4.8) | 4.26 (3.79–4.77) | 521 | 15.2 (13.9–16.6) | 1.12 (1.03–1.22) |
| Other immunosuppresive: yes | | 9 | 5.3 (2.6–9.4) | 4.88 (2.23–9.27) | 26 | 33.6 (21.4–46.3) | 2.43 (1.59–3.57) |

Abbreviations: CI, confidence interval; DOAC, Direct oral anticoagulants; NSAID, Non-steroidal anti-inflammatory drug; SIR, standardized incidence ratio

^a^ Observed cases

### **FIGURE S1.** Flowchart of included study participants

First-time VTE diagnosis during 1996–2022

N = 166,632

Excluded: <18 years of age

N = 737

Age of 18 years or above

N = 165,895

Excluded: no history of migraine

N = 156,524

Previous migraine diagnosis/prescription

N = 9371

Excluded: patients with previous diagnosis of cancer

N = 2240

Study cohort

N = 7131
